# The Prescriber's Pharmacopœia: Containing All the Medicines in the London Pharmacopœia Arranged in Classes According to Their Action, with Their Composition and Doses

**Published:** 1841-07

**Authors:** 


ART. IV.—*The Prescriber's Pharmacopœia: containing all the Medicines in the London Pharmacopœia arranged in classes according to their action, with their composition and doses.* By a Practising Physician.—*London*, 1841. Small 18mo, pp. 126.

THE title-page of this little book indicates its general character; and the author's advertisement explains so well his reasons for compiling it and his grounds for thinking it likely to be useful, that we transcribe the greater portion of it:

"As this little work had its origin in a want daily experienced by the compiler in his practice, he believes that it will be useful to a numerous class of practitioners who possess a memory of the same general character as his own. He has always had great difficulty in calling to mind, at will, a number of heterogeneous particulars which had not been originally contemplated in a systematic order, although he might be well acquainted with them individually. This difficulty he has often painfully experienced when attempting to pass in review the various drugs and officinal formulæ suited to fulfil any special indication that presented itself in the treatment of a disease: and he has frequently witnessed, in the practice of others, a barrenness of prescription and a consequent confined range of resources, originating in the same cause, which could not fail to be injurious to the patient and the reputation of the physician. It is expected that the habit of viewing the articles of the Materia Medica in some such order as that adopted in the present compilation, will tend, in some degree at least, to remedy these evils.

"It is further believed that practitioners, from forgetfulness of the officinal preparations, are often led to prescribe extemporaneous formulæ while there exist better substitutes in the pharmacopœia; and it is hoped that the marshalling together all the various compound forms of medicine under the title of the main ingredient, as in these tables, will at once facilitate and simplify the labours of the prescriber."

We shall be very much mistaken if the author does not find his expectations fulfilled, as we believe that the class of doctors with bad memories comprehends many beyond the pale of the College of Physicians. We extract the first article in the volume to show the manner in which the compiler treats his subject.

ALOE. (A. Spicata. The inspissated juice.)

1. Extractum Aloës purificatum.

*Dose.* Gr. ss–x–xv.

2. Pulvis Aloës compositus.

*Composition.* 3 parts aloes, 1 guaiacum, 2 pulvis cinnam. comp.

*Dose.* Gr. v–℈j.

*Form.* Powder, pill.

3. Pilulæ Aloës compositæ.

*Comp.* 2 aloes, 1 extract of gentian, ol. carui et syrup. q. s.

*Dose.* Gr. j–x–℈j.

4. Pilulæ Aloes cum Myrrha.

*Comp.* 2 aloes, 1 saffron, 1 myrrh.

*Dose.* Gr. j–x–xx.

5. Pilulæ Cambogiæ compositæ.

*Comp.* 3 aloes, 2 camboge, 1 ginger, 4 soap.

*Dose.* Gr. iij–x–xx.

6. Pilulæ Sagapeni compositæ.

*Comp.* 16 sagapenum, 1 aloes, syrup of ginger q. s.

*Dose.* Gr. v–x–℈j.

7. Decoctum Aloës compositum.

*Comp.* Extract of liquorice ʒvij, carbonate of potass ʒj, aloes ʒjss, myrrh ʒjss, saffron ʒjss, water Ojss (boil down to Oj), compound tinct. of cardam. ℥vij.

*Dose.* ℥ss–ij.

*Form.* In other fluids, or simple.

*Incompatible.* Acids, salts, &c. (See Carbonate of Potass.)

8. Tinctura Aloës.

*Comp.* Aloes ℥j, extract of liquorice ℥iij, water Ojss, rectified spirit Oss.

*Dose.* ʒij–℥j.

*Form.* Single, or in other fluids.

9. Tinctura Aloës composita.

*Comp.* Aloes ℥iv, saffron ℥ij, tincture of myrrh, Oij.

*Dose.* ʒj–ij.

*Form.* In other liquids, with other purgatives.

10. Vinum Aloës.

*Comp.* Aloes ℥ij, canella ℥ss, sherry Oij.

*Dose.* ℥ss–ij. (Stomachic ʒj–ij).

*Form.* Simple, or in composition.

11. Enema Aloës.

*Comp.* Aloes ℈ij, carbonate of potass gr. xv, decoction of barley Oss.

*Dose.* Oss. (Enema for ascarides, &c.)

We are always so glad of an opportunity of recommending to our readers the non-restraint system of treating the insane, that we cannot resist the pleasure of transcribing the dedication to the Pharmacopœia, containing, as it does, so just a tribute to one of the highest ornaments of our profession.

"To John Conolly, Resident Physician of the Hanwell Asylum, In sincere admiration and grateful acknowledgment of his noble and successful efforts to ameliorate the condition of the poor Lunatic, by the substitution of a rational system of moral discipline for mechanical restraint, and parental care and kindness for violence and brute force, this little work is, on public grounds, inscribed by the Author."

